# The effect of e-health interventions promoting physical activity in older people: a systematic review and meta-analysis

**DOI:** 10.1186/s11556-020-00239-5

**Published:** 2020-04-21

**Authors:** Rick Yiu Cho Kwan, Dauda Salihu, Paul Hong Lee, Mimi Tse, Daphne Sze Ki Cheung, Inthira Roopsawang, Kup Sze Choi

**Affiliations:** 1 Centre for Gerontological Nursing, School of Nursing, The Hong Kong Polytechnic University, GH502, 5/F, Block G, Hung Hom, Kowloon, Hong Kong, China; 2 School of Nursing, The Hong Kong Polytechnic University, Hong Kong, China; 3 Ramathibodi School of Nursing, Faculty of Medicine Ramathibodi Hospital, Mahidol University, Bangkok, Thailand

**Keywords:** Physical activity, E-health, Older people, Step count, Physical activity energy expenditure

## Abstract

**Introduction:**

The objectives of this review paper were to synthesize the data from randomized controlled trials in the literature to come to a conclusion on the effects of e-health interventions on promoting physical activity in older people.

**Methods:**

The Medline, CINAHL, Embase, PsycINFO, and SportDiscus databases were searched for articles about studies that 1) recruited subjects with a mean age of > 50 years, 2) tested e-health interventions, 3) employed control groups with no or less advanced e-health strategies, 4) measured physical activity as an outcome, 5) were published between 1st January 2008 and 31st May 2019, and 6) employed randomized controlled trials. The risk of bias in individual studies was assessed using the Physiotherapy Evidence Database scale. To examine the effects of the interventions, variables quantifying the amount of physical activity were extracted. The within-group effects of individual studies were summarized using Hedges g and 95% confidence intervals. Between-group effects were summarized by meta-analyses using RevMan 5.0 with a random effect model.

**Results:**

Of the 2810 identified studies, 38 were eligible, 25 were included in the meta-analyses. The within-group effect sizes (Hedges g) of physical activity in the intervention group at T1 ranged from small to large: physical activity time (0.12 to 0.84), step counts (− 0.01 to 11.19), energy expenditure (− 0.05 to 0.86), walking time (0.13 to 3.33), and sedentary time (− 0.12 to − 0.28). The delayed effects as observed in T2 and T3 also ranged from small to large: physical activity time (0.24 to 1.24) and energy expenditure (0.15 to 1.32). In the meta-analysis, the between-group effect of the e-health intervention on physical activity time measured by questionnaires, physical activity time measured by objective wearable devices, energy expenditure, and step counts were all significant with minimal heterogeneity.

**Conclusion:**

E-health interventions are effective at increasing the time spent on physical activity, energy expenditure in physical activity, and the number of walking steps. It is recommended that e-health interventions be included in guidelines to enhance physical activity in older people. Further studies should be conducted to determine the most effective e-health strategies.

## Introduction

Physical activity is defined as any bodily movement produced by skeletal muscles that results in an expenditure of energy [1]. Physical activity is widely recognized as an effective intervention for reducing mortality and dependence-inducing diseases (e.g., cardiovascular disease, cancers) in older people [2]. Studies have shown that engaging in high-intensity aerobic exercise and 150 min of moderate-intensity exercise promotes cognition in older people with mild cognitive impairment [3, 4]. The evidence shows that sustainable physical activity at beneficially high levels of intensity is an important element of improved cognitive function. A systematic review of 39 studies showed that physical activity improved the cognitive function of the older participants regardless of their cognitive status [5]. Another systematic review of nine studies showed that for older people physical activity led to improvements in frailty syndrome, body composition, as well as in the performance of many physical functions (e.g., balance, muscle strength) [6].

Physical inactivity, which is associated with an increased risk of morbidity, mortality, and functional dependence, refers to less than 150 min per week of moderate-to-vigorous physical activity (MVPA) [7]. Physical inactivity remains a prevalent global phenomenon, although the beneficial effect of physical activity is known [8]. Unsurprisingly, the prevalence of physical inactivity increases significantly with age, with the proportion of physically inactive older adults being at 67% globally as reported in a systematic review [9]. Older people were less likely than younger people to engage in regular physical activity [10]. Older people have difficulties achieving the levels of intensity and duration of physical training known to be beneficial [11]. Common barriers to doing so that have been reported in the literature include poor health, a lack of company, lack of interest, lack of skills, and lack of opportunities [12]. Studies have shown that sedentary time (e.g., too much sitting) is also associated with dependence in older people, which is independent of moderate-intensity physical activity [13]. A systematic review showed that even a low dose of moderate-to-vigorous physical activity reduces mortality by 22% in older people [14]. Therefore, the recent evidence shows that it may be more realistic to reduce the amount of time spent in sedentary activities and increase engagement in light activities to pave the way for older people to engage in more intense exercise [11].

Behavioural change interventions are based on a group of psychosocial theories (e.g., social cognitive theory, the transtheoretical model) that posit that people’s behaviours are modifiable when certain factors (e.g., lack of opportunities, lack of skills) are modified [15]. The evidence from many systematic reviews indicates that behavioural change interventions using different behavioural change techniques are effective at motivating different groups of people (e.g., children, obese adults) to increase their levels of physical activity [16, 17]. However, the size of the effect of conventional behavioural change interventions that are delivered face-to-face is suboptimal in older people (d = 0.14), suggesting that many behavioural change techniques that are effective in young people are not effective in older people [18].

E-health refers to health services that are delivered or enhanced through electronic devices, the internet, and related digital technology [19]. Persuasive technology refers to the use of technology designed to guide users into changing particular attitudes and behaviour, by enhancing the effects of the behavioural change techniques [20]. Persuasive technology employed through electronic devices and internet platforms as a form of e-health intervention was recently used to encourage older people to increase their level of physical activity [21]. E-health interventions have been used extensively in dieting interventions and in interventions to promote physical activity in children and young adults, with promising results, as shown in systematic reviews [20, 22–24]. E-health interventions have also been implemented among older people, and their effects on promoting physical activity have been evaluated in clinical trials. A few systematic reviews have shown that many of them employed different e-health strategies, and many individual trials have shown that many e-health interventions are effective at increasing physical activity but some are not [25, 26]. The number of trials included in these reviewers was small and therefore the effects of e-health interventions were not concluded in these reviews.

To date, in the current literature, there is a lack of understanding of the effects of e-health intervention on physical activity in older people because the results from different trials were inconsistent and previous systematic reviews could not conclude the effects with a small number of studies identified. Therefore, this review aimed to add knowledge to the literature about the effects by pooling the data reported in the randomized controlled trials. Specifically, the objectives of this study were to identify:

1. The within-group effect of the e-health interventions on physical activity, and
2. The between-group effect of the e-health interventions on physical activity.

## Methods

A systematic review was employed to identify randomized controlled trials evaluating the effects of e-health interventions on promoting physical activity in older people. The reporting format of this systematic review follows the Preferred Reporting Items for Systematic review and Meta-Analysis (PRISMA) guideline [27].

### Eligibility criteria

- Population: older people (mean age of the sample > 50 years)
- Intervention: e-health intervention, as defined as using any forms of electronic devices, the internet, and related digital technology to promote health service [19]. In this paper, the health service refers to physical activity promotion.
- Control: not exposed to any e-health interventions or to less advanced e-health interventions
- Outcome: physical activity, as defined as either primary or secondary outcome
- Study design: randomized controlled trial
- Language: English

### Sources of information

We searched the following five databases: Medline, CINAHL, Embase, PsycINFO, and SportDiscus. The databases were searched during the period of 1 January 2019 to 31 May 2019.

### Search

Keywords employed for the search included [“older people” or “older adult” or “elderly” or “senior”] **AND** [“texting” or “SMS” or “text messaging” or “mobile device” or “mobile health” or “m-health” or “mHealth” or “e-health” or “eHealth” or “internet-based” or “web-based” or “online” or “DVD-based” or “smartphone” or “mobile phone” or “wearable” or “social media” or “computer” or “tablet”] **AND** [“physical activity” or “exercise” or “step*” or “energy expenditure” or “sedentary”]

In the search engines we limited the results to publications with [abstracts] those published during the period of [1 January 2008–31 may 2019] and those with a study design employing [a randomized controlled trial]

We also conducted a hand search to identify potentially eligible articles by checking relevant article references (e.g., eligible articles and relevant systematic reviews) [28].

### Study selection

Identified articles were imported into Clarivate Analytics Endnote X8.0. Duplicates were removed by Endnote, and then by screening the titles, abstracts, and full texts of the articles. The screening of the articles was conducted by two independent authors. In cases where the two authors disagreed over the eligibility of an article, they discussed the article in relation to the eligibility criteria. If they still disagreed, a third author was invited to discuss the issues over with the two authors to ensure that the article fulfilled the eligibility criteria.

### Data collection process

Data were extracted from the full texts of the eligible articles. The selected items of data were copied to a piloted form using Microsoft Excel. Data extraction was conducted by two authors independently. If there were any disagreements over the extraction of data, the two authors invited the third author to discuss the matter according to the pre-defined nature of the data items. In the case of queries, attempts were made to contact the authors of the studies for clarification.

### Data items

To describe the profile of the articles, the following data were extracted: authors, year of publication, age of the subjects (mean and standard deviation), sample size, population characteristics, intervention, controlled condition, outcome, data collection time points, e-health strategies, and targeted physical activity.

To examine the effect of the intervention on the outcome, all variables quantifying the amount of physical activity were extracted (e.g., time spent on physical activity, energy expended on physical activity, step counts, sedentary time). Also extracted were the values of the outcome variables (i.e., mean, standard deviation, and sample size in each group) observed at the baseline (T0), the time point after the completion of the intervention (T1), and the 1st (T2) and 2nd (T3) follow-ups after the completion of the intervention in both the intervention groups and control groups.

### Risk of bias in individual studies

This review employed the Physiotherapy Evidence Database (PEDro) scale to rate the quality of RCTs [29]. The PEDro scale is comprised of 11 dichotomous items (i.e., yes/no) measuring the methodological quality of an RCT (e.g., blinding, concealment, random allocation, baseline similarity, dropout). Except for the first item (i.e., specified eligibility criteria), all 10 items sum up to a total score. The quality of the RCT is rated as excellent (PEDro = 9–10), good (PEDro = 6–8), fair (PEDro = 4–5), or poor (PEDro< 4). We considered studies with a PEDro score of ≥4 to have a minimal standard of methodological quality, and we therefore included only those studies in the quantitative synthesis (i.e., meta-analysis of the effects).

### Summary measures and synthesis of the results

We followed the Cochrane Handbook for Systematic Results of individual studiesReviews of Interventions to handle and analyse the data to run a meta-analysis [30]. Both within-group and between-group effects (i.e., T1 between the intervention and control groups) of individual studies were summarized using Hedges g and a 95% confidence interval.

A meta-analysis was performed if three or more studies measured the same outcome and the articles provided the mean and standard deviation of the outcome variables at T1 (i.e., the time point immediately after the completion of the intervention), in order to understand the immediate between-group effects. A subgroup analysis of the same outcome measured by objective instruments (e.g., pedometers, accelerometers) and subjective instruments (e.g., questionnaires) was conducted separately to minimize heterogeneity among the studies. The results of the meta-analysis are presented through Forest plots using RevMan version 5.0. The I^2^ index was used to test the heterogeneity of the selected studies. We report a meta-analysis on the outcomes with heterogeneity, which might not be important (i.e., I^2^ = 0–40%), only to ensure the quality of the interpretation of the pooled effects [31]. Random effect models were used because the intervention components in the selected studies were not identical [32], although in all of the studies e-health strategies were used in the interventions.

## Results

### Study selection

**Fig. 1:**
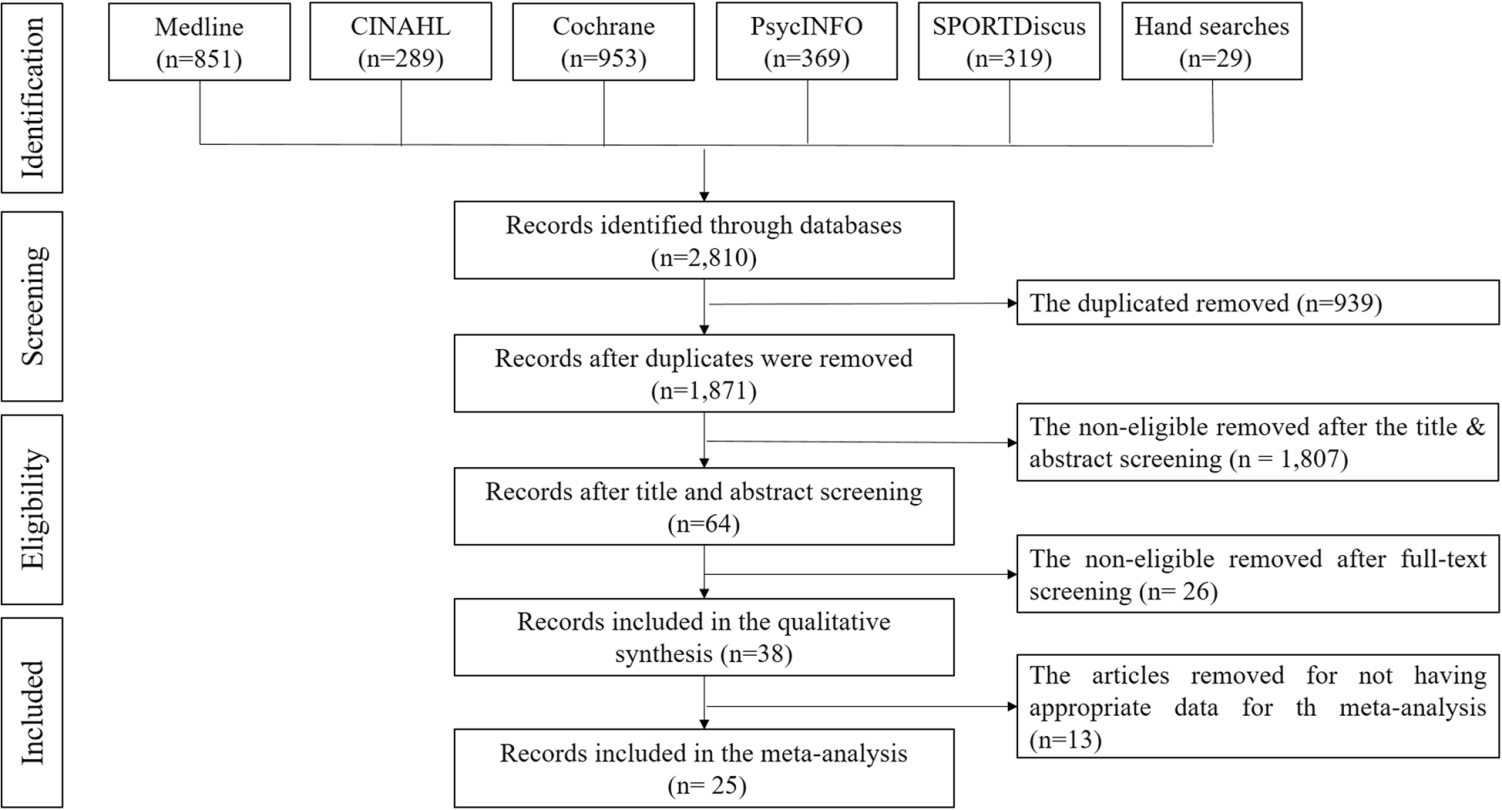
Prisma flowchart

As shown in Fig. 1, 2,810 articles were identified in the selected databases: Medline (*n* = 851), CINAHL (*n* = 289), Cochrane (*n* = 953), PsycINFO (*n* = 369), SPORTDisuc (*n* = 319), and a hand search (*n* = 29). Nine hundred and thirty-nine articles were removed by Endnote and manual screening because they were duplicates, 1807 were removed after screening for title and abstract because they were not eligible, and 26 were removed for ineligibility after a full-text screening. Thirty-eight articles were eligible for a qualitative synthesis. After the extraction of data, 13 articles were not included in the meta-analysis because the risk of bias as rated by the PEDro score was high (*n* = 2) [33, 34], the mean and standard deviation at T1 of both groups were not provided (*n* = 4) [35–38], the effect size or standard deviation were outlined (*n* = 3) [39–41], the outcome variables were measured by fewer than three studies (*n* = 3) [42–44], and the data were from a preliminary analysis, which duplicated data in another study reporting the final analysis (*n* = 1) [45]. In the end, 25 articles were included in the meta-analyses of different outcomes.

### Study characteristics

As shown in Table 1, 38 eligible articles were on randomized controlled trials evaluating the effects of e-health interventions on physical activity outcomes over a total population of 11,194 people, whose mean age ranged from 50.8 to 82 years. The majority of the studies targeted healthy (*n* = 25, 65.8%), physically inactive (*n* = 21, 55.5%) older people. Apart from healthy subjects, the remaining studies recruited subjects with different health conditions, including obesity/overweight, cardiac diseases, COPD, obstructive sleep apnoea, diabetes, rheumatoid arthritis, Parkinson’s disease, and cancer.

**Table 1:** Profile of the selected articles

No	1st Author	Year	Sample size	Age Mean (SD)	Population	Intervention	Control	Outcomes	Time
1	Pinto [46]	2005	*N* = 100	I:68.5 C:68.5	Healthy PI	PA: non-specific EH: tele-counselling	F2f PA advice	PA time EE PA unit	T1:3m T2:6m
2	King [47]	2007	*N* = 218	C1:60.2 (4.5) C2: 60.5 (6.0) I:61.6 (5.9)	Healthy PI	PA: non-specific EH: automated advice	C1: F2f PA advice C2: F2f HE	EE PA time	T1:6m T2:12m
3	Kolt [48]	2007	*N* = 186	I:74.1(6.2) C:74.3(5.9)	Healthy PI	PA: non-specific EH: tele-counselling	Usual care	PA time Walk time	T1:3m T2:6m T3:12m
4	King [49]	2008	*N* = 37	I: 60.7 (6.8) C: 59.6 (7.6)	Healthy PI	PA: non-specific EH: digital PA recording	Written HE	PA time EE	T1:8w
5	Martinson [45]	2008	*N* = 1049	57.1(0.2)	Healthy PI	PA: non-specific EH: tele-counselling, PA auto-tracking feedback	Usual care	EE	T1:6m
6	Laubach [50]	2009	*N* = 30	I: 63.9 (4.1) C:64.9 (4.1)	Healthy	PA: walking EH: PA auto-tracking feedback	Usual care	Step count	T1:8w
7	Martinson [51]	2010	*N* = 1049	57.1(0.2)	Healthy PI	PA: non-specific EH: tele-counselling, PA auto-tracking feedback	Written HE	EE	T1:6m T2:12m T3:24m
8	Kahlbaugh [34]	2011	*N* = 35	82 (9.8)	Healthy	PA: non-specific EH: Video game	C1: TV watching C2: usual care	PA unit	T1:10w
9	Van Stralen [52]	2011	*N* = 1971	64 (8.6)	Healthy	PA: non-specific EH: digital-tailored advice	C1: digital-tailored advice (reduced form) C2: usual care	PA time	T1:12m
10	Peels [35]	2013	*N* = 1248	I: 61.6 (7.8) C1: 63.2 (8.3) C2: 63.7 (8.9) C3: 62.6 (7.2) C4: 64.1 (9.0)	Healthy	PA: non-specific EH: digital-tailored advice	C1: written advice C2: electronic advice C3: written advice C4: Usual care	PA time	T1:3m T2:6m T3:12m
11	Bickmore [53]	2013	*N* = 263	71.3 (5.4)	Healthy PI	PA: walking, stretching EH: digital-tailored advice, video demonstration	PA tracking	Step count	T1:12m
12	Irvine [54]	2013	*N* = 368	60.3(4.9)	Healthy PI	PA: endurance, stretching, strengthening, & balance EH: digital-tailored advice	Usual care	PA time PA frequency	T1:12w T2:24w
13	King [55]	2013	*N* = 40	68.3 (8.2)	Healthy PI	PA: walking, stretching EH: digital-tailored advice, video demonstration	F2f HE	Step count Walk time	T1:4m
14	Wijsman [36]	2013	*N* = 226	I: 64.7 (3.0) C: 64.9 (2.8)	Healthy PI	PA: non-specific EH: PA auto-tracking feedback, digital PA coaching	Usual care	PA time	T1:3m
15	Kim [56]	2013	*N* = 36	I: 70.6 (7.5) C: 69.3 (7.3)	Healthy	PA: non-specific EH: digital PA coaching	Usual care	Step count PA unit	T1:6w
16	Mendelson [57]	2014	*N* = 107	I:62.0 (9.0) C:63.0 (9.0)	OSA	PA: non-specific EH: tele-counselling	Usual care	Step count EE	T1:4m
17	Tabak-a [58]	2014	*N* = 24	I:64.1(9.0) C:62.8(7.4)	COPD PI	PA: mobilization, resistance, endurance EH: digital PA coaching, tele-counselling, video demonstration	Usual care	PA unit PA count	T1:1m T2:3m
18	Tabak-b [59]	2014	*N* = 32	I:65.2(9.0) C:67.9(5.7)	COPD PI	PA: non-specific EH: digital-tailored advice	Usual care	Step count	T1:1w T2:2w T3:3w
19	Thompson [42]	2014	*N* = 49	I: 79.1(8.0) C: 79.8(6.0)	Healthy PI	PA: endurance, strength, balance, flexibility EH: PA auto-tracking feedback	Usual care	PA unit	T1:6m
20	Vroege [60]	2014	*N* = 235	I:64.7(3.0) C:64.9(2.8)	Healthy PI	PA: non-specific EH: PA auto-tracking feedback, digital PA coaching	Usual care	PA time	T1:3m
21	Frederix [41]	2015	*N* = 140	I:61.0 (9.0) C:61.0 (8.0)	Cardiac diseases	PA: Endurance EH: Digital PA coaching	F2f PA advice	Step count PA time	T1:6w T2:24w
22	Maddison [61]	2015	*N* = 171	I:61.4(8.9) C:59.0(9.5)	Cardiac diseases	PA: walking, household chores and active transport EH: video vignette, automated advice, online resource	Usual care	PA time Walk time	T1:24w
23	Martin [62]	2015	*N* = 48	I:55 (8) C1:58 (8) C2:60(7)	Obese, diabetes, cardiac disease	PA: non-specific EH: PA auto-tracking feedback, digital PA coaching	C1: Unblinded PA tracking + texting C2: Unblinded PA tracking	Step count PA time	T1:1w
24	Mouton (76)	2015	*N* = 149	65.0 (6.0)	Healthy	PA: endurance, strength, balance, flexibility EH: digital tailored advice	C1: centre-based C2: web-based C3: usual care	PA time	T1:12m
25	Van der Weegen [63]	2015	*N* = 199	I:57.5(7.0) C1:56.9(8.3) C2:59.2(7.5)	Diabetes, COPD	PA: non-specific EH: digital PA coaching, PA auto-tracking feedback	C1: f2f support C2: usual care	PA time	T1:4-6m T2:9m
26	Broekhuizen [64]	2016	*N* = 235	I: 64.7 (3.0) C: 64.9 (2.8)	Healthy PI	PA: non-specific EH: PA auto-tracking feedback, digital PA coaching	Usual care	PA time	T1:3m
27	King [37]	2016	*N* = 95	I:57.9(7.7) C1:62.8(9.8) C2:59.5(9.5) C3:59.5(10.0)	Healthy PI	PA: non-specific EH: PA auto-tracking feedback, digital PA coaching	C1: social app C2: affect app C3: usual care	PA time Walk time Sed time	T1:8w
28	Muller [65]	2016	*N* = 43	63.3 (4.5)	Healthy PI	PA: non-specific EH: digital PA coaching	Written HE	PA time Sed time	T1:12w T2:24w
29	Parker [33]	2016	*N* = 28	I: 58.2(6.6) C:61.6(5.5)	Healthy	PA: aerobic PA EH: digital PA coaching	Texting PA reminder	PA time	T1:4w
30	Thakkar [39]	2016	*N* = 710	57.6 (9.2)	Cardiac disease	PA: non-specific EH: digital PA coaching	Cardiac rehabilitation	PA time Sed time	T1:6m
31	Thomsen [43]	2016	*N* = 20	I:64.5(8.5) C:54.0(14.0)	RA PI	PA: non-specific EH: digital PA coaching	Usual care	Sed time	T1:16w
32	Demeyer [66]	2017	*N* = 343	I: 66 (8) C:67 (8)	COPD	PA: non-specific EH: digital PA coaching, PA auto-tracking feedback	Written HE	PA time Step count Walk time	T1:12w
33	Krebs [44]	2017	*N* = 86	59.8 (11.4)	Cancer PI	PA: non-specific EH: digital PA coaching	F2f advice & brief counselling	PA unit	T1:3m
34	Lyons [67]	2017	*N* = 40	61.5 (5.6)	Overweight PI	PA: non-specific EH: PA auto-tracking feedback, digital PA coaching	Usual care	Step count Walk time Sed time	T1:12w
35	Nahm [68]	2017	*N* = 866	62.8 (8.5)	Healthy	PA: non-specific EH: Online HE	Usual care	PA time EE	T1:8w
36	Alley [38]	2018	*N* = 504	50.8 (13.1)	Healthy PI	PA: walking EH: PA auto-tracking feedback, digital PA recording, online social support	C1: pedometer feedback, online recording C2: logbook recording	PA time Step count	T1:3m T2:12m T3:18m
37	Ellis [69]	2019	*N* = 44	I:64.8 (8.5) C1:63.3 (10.6) C2:64.1 (9.5)	Parkinson’s disease	PA: individualized exercise, walking EH: digital PA coaching, PA auto-tracking feedback	F2f counselling, Pedometer feedback	PA time Step count	T1:12m
38	Rowley [70]	2019	*N* = 170	I:67.4 (6.4) C1:66.1 (4.9) C2: 68.3 (7.1)	Healthy PI	PA: walking EH: PA auto-tracking feedback, digital PA coaching	C1: pedometer feedback, logbook recording C2: usual care	Step count	T1:12w

*I*  intervention group, *C* Control group, *PI* Physically inactive, *PA* Physical activity, *PA freq* Physical activity frequency, *VSC day* Valid step count day, *EE* Energy expenditure, w = week, m = month, walk time = walking time, *PD* Parkinson’s Disease, *CHD* Chronic Heart Diseases, *OSA* Obstructive Sleep Apnea, *CR* Cardiovascular Risk, *TV* Television, *COPD* Chronic Obstructive Disease, Sed time = Sedentary time

Most of the interventions did not promote a specific type of physical activity (*n* = 25, 65.8%). Walking was the most common target for the subjects to practise to increase their level of physical activity (*n* = 7, 18.4%). Other forms of physical activity promoted in the interventions included endurance exercises, stretching, flexibility, and balance, mobilization, resistance, and individualized exercise training.

With regard to the controlled conditions, many studies employed more than one control group, while the usual care was the most commonly used form of control (*n* = 23, 60.5%). Other studies used active control strategies, such as using fewer e-health strategies, different types of e-health strategies (e.g., social support apps), or non-digital behavioural change strategies (e.g., face-to-face counselling, face-to-face health education, recording steps on logbooks).

Most of the studies employed physical activity time (*n* = 22, 57.9%) to quantify amounts of physical activity. Other methods were also used to measure physical activity, including step count (*n* = 13, 34.2%), energy expenditure (*n* = 10, 26.3%), walking time (*n* = 7, 18.4%), sedentary time (*n* = 5, 13.2%), physical activity units calculated by a specific physical activity measuring instruments (*n* = 6, 15.8%), and physical activity frequency (*n* = 1, 2.6%).

Most of the studies did not conduct follow-up measurements after T1 (*n* = 23, 60.5%). The T1 observation time points were from 1 week to 12 months away from the baseline. The post-T follow-up time points were from 2 weeks to 24 months away from the baseline.

Different e-health strategies were identified in the interventions. As shown in Table 2, 11 e-health strategies were used in the identified studies: 1) automated advice (*n* = 2), 2) tele-counselling (*n* = 6), 3) digital-tailored advice (*n* = 7), 4) digital physical activity recording (*n* = 2), 5) digital physical activity coaching (*n* = 18), 6) online resources (*n* = 2), 7) online social support (*n* = 1), 8) physical activity auto-tracking feedback (*n* = 15), 9) video demonstrations (*n* = 3), 10) video games (*n* = 1), and 11) video vignettes (*n* = 1). Many studies employed multiple e-health strategies concurrently to develop their interventions. The categories are not mutually exclusive. For example, in some studies digital physical activity coaching also included online social support and digital-tailored advice. Earlier studies tended to use fewer e-health strategies, while later studies tended to use more.

**Table 2:** E-health intervention strategies

E-health strategies	Description
1. Automated advice	Provide pre-designed physical activity advice (e.g., benefits of physical activities) to participants automatically by computer or internet.
2. Tele-counselling	Provide physical activity counselling (e.g., goal-setting, prompting, planning) by human facilitators via telephone or smartphone.
3. Digital-tailored advice	Provide physical activity advice (e.g., time, types, benefits of physical activity) to participants considering the participants’ individuality (e.g., baseline physical activity) by computer or internet.
4. Digital PA recording	Allow participants to input their physical activity performance (e.g., step count) so that participants can understand the progress of their performance.
5. Digital PA coaching	Providing coaching (e.g., goal setting, prompting, social support, demonstrations) for participants via digital platforms (e.g., online forums, texting) according to the individuality of the participants (e.g., baseline physical activity performance, on-going progress).
6. Online resources	Provide physically active lifestyle resources online (e.g., types of physical activity, health benefits of physical activities, places to perform physical activity).
7. Online social support	Provide an online platform for participants and the facilitator to share their physical activity tips and supportive messages.
8. PA auto-tracking feedback	Provide automatic tracking and feedback (e.g., trend of step counts, physical activity time, percentage of target achieved) by wearable devices (e.g., smartphones, wrist bands).
9. Video demonstrations	Provide physical activity demonstrations via video (e.g., DVD, online video streaming).
10. Video games	Provide video-game-based activities to enhance physical activity time.
11. Video vignettes	Provide successful stories of behavioural change from being sedentary to becoming physically active.

Digital physical activity coaching was the most widely adopted method (*n* = 18, 47.3%). Multiple behavioural change techniques were employed in the digital physical activity coaching reported in the studies, including setting goals, giving out rewards, making demonstrations, and extending social support. These techniques were implemented on various digital platforms such as text messaging platforms, websites, DVDs, PDAs, and email. Physical activity auto-tracking feedback was the second most adopted method as reported in the identified articles (*n* = 15, 39.5%). The strategy involves instructing the subjects to wear accelerometer- or pedometer-embedded wearable devices (e.g., smartphones, wrist-worn devices) to track their physical activity levels, and giving feedback to the subjects automatically in terms of graphs or figures that are meaningful to the subjects (e.g., step counts, percentage of physical activity goals achieved).

### Risk of bias within studies

As shown in Table 3, the PEDro total scores of the 38 articles ranged from 2 to 8. Twenty articles (52.6%) were rated as good, sixteen (42.1%) as fair, and two (5.3%) as poor in quality.

**Table 3:** Risk of bias in individual studies using the PEDro scale

No	Authors	Year	Eligibility	Random allocation	Concealed	Baseline similarity	Blinding (P)	Blinding (T)	Blinding (A)	Dropout	ITT	Group comparison	Point measures and variability data	PEDro total score	Quality rating
1	Pinto et al.	2005	Yes	Yes	No	Yes	No	No	No	Yes	Yes	Yes	Yes	6/10	Good
2	King et al.	2007	Yes	Yes	No	Yes	No	No	Yes	Yes	Yes	Yes	Yes	7/10	Good
3	Kolt et al.	2007	Yes	Yes	No	Yes	No	No	Yes	Yes	No	Yes	Yes	6/10	Good
4	King et al.	2008	Yes	Yes	No	Yes	No	No	No	Yes	No	Yes	Yes	5/10	Fair
5	Martinson et al.	2008	Yes	Yes	Yes	Yes	No	No	No	Yes	No	Yes	Yes	6/10	Good
6	Laubach et al.	2009	Yes	Yes	No	No	No	No	No	Yes	Yes	Yes	Yes	5/10	Fair
7	Martinson et al.	2010	Yes	Yes	Yes	Yes	No	No	No	Yes	Yes	Yes	Yes	6/10	Good
8	Kahlbaugh et al.	2011	Yes	Yes	No	No	No	No	No	No	No	No	No	2/10	Poor
9	Van Stralen et al.	2011	Yes	Yes	No	Yes	No	No	No	No	Yes	Yes	Yes	5/10	Fair
10	Peels et al.	2013	Yes	Yes	No	Yes	No	No	No	No	No	Yes	Yes	4/10	Fair
11	Bickmore et al.	2013	Yes	Yes	No	No	No	No	Yes	Yes	Yes	Yes	Yes	6/10	Good
12	Irvine et al.	2013	Yes	Yes	No	No	No	No	No	No	Yes	Yes	Yes	4/10	Fair
13	King et al.	2013	Yes	Yes	No	Yes	No	No	Yes	Yes	Yes	Yes	Yes	7/10	Good
14	Wijsman et al.	2013	Yes	Yes	Yes	Yes	No	No	No	Yes	Yes	Yes	Yes	7/10	Good
15	Kim & Glanz	2013	Yes	Yes	No	Yes	No	No	No	No	Yes	Yes	Yes	5/10	Fair
16	Mendelson et al.	2014	Yes	Yes	No	Yes	No	No	No	No	Yes	Yes	Yes	5/10	Fair
17	Tabak et al.	2014	No	Yes	Yes	No	No	No	No	No	Yes	Yes	Yes	5/10	Fair
18	Tabak et al.	2014	Yes	Yes	No	Yes	No	No	No	Yes	No	Yes	Yes	5/10	Fair
19	Thompson et al.	2014	Yes	Yes	No	Yes	No	No	No	Yes	No	Yes	Yes	5/10	Fair
20	Vroege et al.	2014	Yes	Yes	Yes	Yes	No	No	Yes	Yes	Yes	Yes	Yes	8/10	Good
21	Frederix et al.	2015	Yes	Yes	No	Yes	No	No	Yes	Yes	Yes	Yes	Yes	7/10	Good
22	Maddison et al.	2015	Yes	Yes	Yes	Yes	No	No	Yes	Yes	Yes	Yes	Yes	8/10	Good
23	Martin et al.	2015	Yes	Yes	No	Yes	No	No	No	Yes	Yes	Yes	Yes	6/10	Good
24	Mouton et al.	2015	Yes	Yes	No	Yes	No	No	Yes	No	No	Yes	Yes	5/10	Fair
25	Van de Weegen et al.	2015	Yes	Yes	Yes	Yes	No	No	No	Yes	Yes	Yes	Yes	5/10	Good
26	Broekhuizen et al.	2016	Yes	Yes	Yes	Yes	No	No	Yes	Yes	Yes	Yes	Yes	8/10	Good
27	King et al.	2016	Yes	Yes	No	Yes	No	No	No	Yes	No	Yes	Yes	5/10	Fair
28	Muller et al.	2016	Yes	Yes	Yes	Yes	No	No	No	Yes	Yes	Yes	Yes	7/10	Good
29	Parker et al.	2016	Yes	Yes	No	No	No	No	No	No	No	Yes	Yes	3/10	Poor
30	Thakkar et al.	2016	Yes	Yes	Yes	Yes	No	No	No	Yes	No	Yes	Yes	6/10	Good
31	Thomsen et al.	2016	Yes	Yes	No	Yes	No	No	Yes	Yes	No	Yes	Yes	6/10	Good
32	Demeyer et al.	2017	No	Yes	Yes	Yes	No	No	No	Yes	Yes	Yes	Yes	7/10	Good
33	Krebs et al.	2017	Yes	Yes	No	Yes	No	No	No	No	No	Yes	Yes	4/10	Fair
34	Lyons et al.	2017	Yes	Yes	Yes	Yes	No	No	No	Yes	Yes	Yes	Yes	7/10	Good
35	Nahm et al.	2017	Yes	Yes	No	Yes	No	No	No	No	Yes	Yes	Yes	5/10	Fair
36	Alley	2018	Yes	Yes	No	Yes	No	No	No	No	No	Yes	Yes	4/10	Fair
37	Ellis et al.	2019	Yes	Yes	Yes	Yes	No	No	Yes	Yes	No	Yes	Yes	7/10	Good
38	Rowley et al.	2019	Yes	Yes	No	Yes	No	No	No	No	No	Yes	Yes	4/10	Fair

*ITT* Intention-to-treat

### Objective 1: identify the within-group effect of the interventions on physical activity

As shown in Table 4, the within-group effect size (Hedges G) of physical activity time in the intervention group at T1 ranged from 0.12 to 0.84, step counts from − 0.01 to 11.19, energy expenditure from − 0.05 to 0.86, walking time from 0.13 to 3.33, sedentary time from − 0.12 to − 0.28, physical activity units from − 0.41 to 1.86, and physical activity frequency at 0.84. The delayed effects as observed in T2 and T3 on physical activity time ranged from 0.24 to 1.24, and on energy expenditure from 0.15 to 1.32.

**Table 4:** Results of individual studies

No.	Author/Year	Outcome	Measurement	Effect Size – within group (Hedges G)		
				T1	T2	T3
1	Pinto 2005 [46]	PA time EE PA unit	7-Day PAR (min/week) 7-Day PAR (kcal/day) Accelerometer (count)	0.58 0.60 0.43	0.71 0.72 0.36	
2	King 2007 [47]	EE PA time EE PA time	CHAMPS (kcal/kg/day) CHAMPS (time/week) 7-Day PAR (kcal/kg/day) 7-Day PAR (min/week)	0.86 0.84 0.66 0.79	1.32 1.24 0.64 0.62	
3	Kolt 2007 [48]	PA time Walk time	AHSPAQ (min/week) AHSPAQ (min/week)	0.18 0.40	0.24 0.19	0.35 0.21
4	King 2008 [49]	PA time EE	CHAMPS (min/week) CHAMPS (kcal/kg/week)	0.77 0.69		
5	Martinson 2008 [45]	EE	CHAMPS (kcal/week)	−0.03		
6	Laubach 2009 [50]	Step count	Pedometer (step/day)	0.50		
7	Martinson 2010 [51]	EE	CHAMPS (kcal/week)	0.07	0.15	0.17
8	Kahlbaugh 2011 [34]	PA unit	WPAS (score)	NA		
9	Van Stralen 2011 [52]	PA time	SQUASH (min/week)	0.17		
10	Peels 2013 [35]	PA time	SQUASH (min/week)	NA		
11	Bickmore 2013 [53]	Step count	Pedometer (step/day)	0.01		
12	Irvine 2013 [54]	PA time PA frequency	SDQ (min/week) SDQ (count/week)	NA 0.84	NA 0.79	
13	King 2013 [55]	Step count Walk time	Pedometer (step/day) CHAMPS (min/week)	NA 3.44		
14	Wijsman 2013 [36]	PA time	Accelerometer (min/day)	NA		
15	Kim 2013 [56]	Step count PA unit	Pedometer (step/day) LTEQ (score)	0.29 1.86		
16	Mendelson 2014 [57]	Steps count EE	Accelerometer (step/day) Accelerometer (kcal/week)	−0.06 − 0.05		
17	Tabak 2014 [58]	PA unit PA unit	BPAQ (score) Accelerometer (count/min)	−0.41 0.13	0.07 −0.16	
18	Tabak 2014 [59]	Step count	Accelerometer (step/day)	0.09	0,14	−0.05
19	Thompson 2014 [42]	PA unit	Accelerometer (unit/day)	−0.14		
20	Vroege 2014 [60]	PA time	Accelerometer (min/day)	0.60		
21	Frederix 2015 [41]	PA time Step count	IPAQ (min/week) Accelerometer (step/day)	NA 11.19^a^	NA 27.6^a^	
22	Maddison 2015 [61]	PA time Walk time	IPAQ (min/week) IPAQ (min/week)	0.17 0.13		
23	Martin 2015 [62]	Step count PA time	Accelerometer (steps/day) Accelerometer (min/day)	0.39 0.71		
24	Mouton 2015 [71]	PA time	IPAQ (min/week)	0.33		
25	Van de Weegen 2015 [63]	PA time	Accelerometer (min/day)	0.75	0.76	
26	Broekhuizen 2016 [64]	PA time	Accelerometer (min/day)	0.59		
27	King 2016 [37]	PA time Sed time Walk time	Accelerometer (min/day) Accelerometer (min/day) Accelerometer (min/day)			
28	Muller 2016 [65]	PA time Sed time	IPAQ-S (min/week) IPAQ-S (hr/day)	0.75 −0.12	0.85 −0.03	
29	Parker 2016 [33]	PA time	EPAP (min/week)	NA		
30	Thakkar 2016 [39]	PA time Sed time	GPAQ(min/week) GPAQ (min/week)	0.82 NA		
31	Thomsen 2016 [43]	Sed time	ActivPAL3 (hours/day)	−0.15		
32	Demeyer 2017 [66]	Step count PA time Walk time	Accelerometer (step/day) Accelerometer (min/day) Accelerometer (min/day)	0.11 0.12 0.19		
33	Krebs 2017 [44]	PA unit	GLTEQ (MET units/week)	−0.16		
34	Lyons 2017 [67]	Step count Walk time Sed time	Accelerometer (step/day) Accelerometer (min/day) Accelerometer (min/day)	0.41 0.58 −0.28		
35	Nahm 2017 [68]	PA time EE	YPAS (min/week) YPAS (kcal/week)	0.21 0.21		
36	Alley 2018 [38]	PA time Step count	Accelerometer (min/day) Accelerometer (step/day)	NA NA		
37	Ellis 2019 [69]	Steps count PA time	Pedometer (step/day) Pedometer (min/day)	0.01 0.13		
38	Rowley 2019 [40]	Steps count	Pedometer (step/day)	2.34^a^		

^a^Outlining effect size, which was excluded from the meta-analysis

*PA* Physical activity, *EE* Energy expenditure, *Sed time* Sedentary time, *CHAMPS* Community Healthy Activities Model Program questionnaire for older adults, *SQUASH* Short questionnaire to assess health enhancing physical activity, *GLTEQ* Godin Leisure Time Exercise Questionnaire, *YPAS* Yale Physical Activity Survey, *EPAP* Electronic Physical Activity Participation Form, *WPAS* Weekly Physical Activity Scale, 7-Day = 7-Day Physical Activity Recall, *AHSPAQ* Auckland Heart Study Physical Activity Questionnaire, *BPAQ* Baecke Physical Activity Questionnaire, *GPAQ* Global Physical Activity Questionnaire; *GPPAQ* General Practice Physical Activity Questionnaire, *SDQ* Self-developed questionnaire

### Objective 2: identify the between-group effect of the interventions on physical activity

**Fig. 2:**
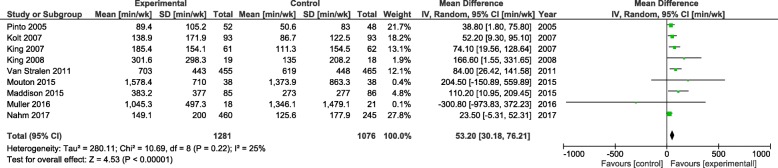
Florest plot of the effect of e-health interventions on phyiscal activity time measured by questionnaires

In the Forest plot shown in Fig. 2, the between-group effect of the e-health intervention on physical activity time measured by questionnaires was analysed by meta-analysis on nine studies that included 2357 subjects. The result showed minimal heterogeneity among the included studies (I^2^ = 25%). The overall effect showed that the interventions led to a significant increase in physical activity time (mean difference = 53.2 min/week, 95%CI = 30.18–76.21) when compared with the result for the control groups.

**Fig. 3:**
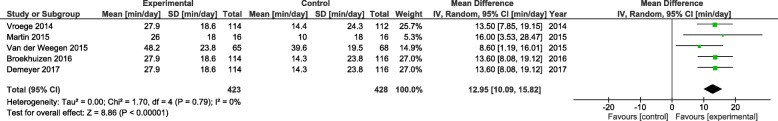
Florest plot of the effect of e-health interventions on physical activity time measured by objective wearable devices

In the Forest plot shown in Fig. 3, the between-group effect of the e-health intervention on physical activity time measured using objective wearable devices (i.e., accelerometers) was analysed by meta-analysis on five studies that included 851 subjects. The result showed negligible heterogeneity among the included studies (I^2^ = 0%). The overall effect showed that the interventions led to a significant increase in physical activity time (mean difference = 12.95 min/day, 95%CI = 10.09–15.82) when compared with the result for the control groups.

**Fig. 4:**
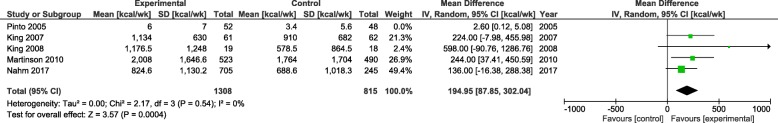
Florest plot of the effect of e-health interventions on energy expenditure

In the Forest plot shown in Fig. 4, the between-group effect of the e-health intervention on energy expenditure was analysed by meta-analysis on four studies that included 2123 subjects. The result showed negligible heterogeneity among the four included studies (I^2^ = 0%). The overall effect showed that the interventions led to a significant increase in energy expenditure (mean difference = 194.95 kcal/week, 95%CI = 87.85–302.04) when compared with the result for the control groups.

**Fig. 5:**
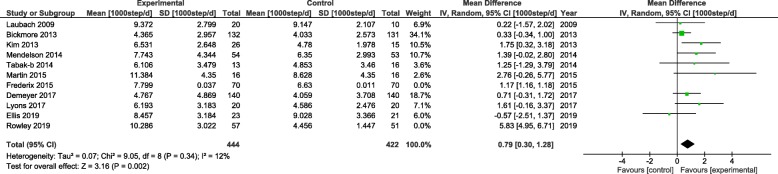
Florest plot of the effect of e-health intervention on step counts

In the Forest plot shown in Fig. 5, the between-group effect of the e-health intervention on step counts measured by objective wearable devices (i.e., accelerometers or pedometers) was analysed by meta-analysis on 11 studies that included 866 subjects. The result showed minimal heterogeneity among the nine included studies (I^2^ = 12%). The overall effect showed that the interventions led to a significant increase in step counts (mean difference = 790step/day, 95%CI = 300–1280) when compared with the result for the control groups.

For the walking time, the between-group effect of the e-health intervention measured by objective wearable devices (i.e., accelerometers or pedometers) was analysed by meta-analysis on three studies that included 345 subjects. However, the heterogeneity was too high to generate a reliable result for the pooled effect on this outcome (I^2^ = 74%). The between-group effect of the e-health intervention on walking time measured by questionnaires was also analysed by meta-analysis on three studies that included 397 subjects. The heterogeneity was also too high (I^2^ = 85%).

For the outcomes of sedentary time (*n* = 2), physical activity unit (*n* = 2), and physical activity frequency (*n* = 1), there were fewer than three studies that measured these outcomes with comparable instruments and valid data. Therefore, meta-analyses of between-group effects were not conducted on these outcomes.

## Discussion

This is the largest systematic review of previously conducted randomized controlled trials using e-health interventions to promote physical activity in older people to come to the conclusion, from a quantitative determination of their effects, that such interventions are effective. They are particularly effective at increasing the time and energy that older people spend on performing physical activities as well as walking. This is also the first study to have systematically summarized 11 e-health strategies that were employed in those trials to enhance older people’s physical activity. These findings have important implications for both clinicians and researchers.

The pooled within-group effect size of the e-health interventions on physical activity time was mild to moderate (d = 0.12–0.84). The effect size was obviously higher than that of conventional behavioural change interventions promoting physical activity in older people as reported in a systematic review (d = 0.14) [18]. This echoes the argument raised in a previous study that conventional behavioural change interventions that have been found to be effective at changing behaviours in younger people may not be as effective in older people [18]. Yet this review supports the view that e-health strategies may be effective at enhancing the effect of conventional behavioural change techniques. A further study should be conducted to test which e-health strategies are more effective at promoting physical activity in older people.

The pooled between-group effect size of the e-health interventions promoting physical activity is seemingly clinically meaningful in authors’ opinion. It is because the participants in the intervention groups had a mean difference of 53.2 more physical activity minutes per week as measured by actigraphs and 90.7 more physical activity minutes per week as measured by questionnaires than those in the control groups. These differences are over 35 and 60% of the physical activity time recommended by WHO as yielding health benefits in older people (i.e., 150 min/week) [8]. Therefore, it is recommended that e-health interventions be included in guidelines for promoting physical activity in older people.

In the subgroup analysis, the effect of e-health interventions on the physical activity time between that measured by actigraphs and that measured by questionnaires was observed to be quite different. The physical activity measured by questionnaires was observed to have a much higher value than that measured by actigraphs. This observation is comparable with what was reported in the literature, namely, that the use of questionnaires likely leads to over-estimations of actual physical activity [72]. In order to more precisely identify the effects of e-health interventions, future studies should adopt objective measurements of physical activity.

Earlier studies showed that the common reasons for older people to avoid performing physical activities are inconvenience and a lack of access to physical activity programmes [68]. This review found that walking is the most commonly targeted physical activity for older people since there are no problems involved with gaining access to programmes, because it is an activity that can be practised anywhere. This review also showed that participants in the e-health intervention groups walked significantly more than those in the control groups (mean difference = 790 steps/day). Walking at a speed of 2.5 km/hr. is sufficient for older people to achieve the intensity of MVPA [73]. Therefore, it is advocated that walking be the physical activity that is targeted for promotion in older people.

Lack of social support and fear of falling were also identified in the literature as common barriers to the participation of physical activity by older people [74]. This review found that online social support is a common e-health strategy to promote physical activity in older people. Studies echoed the view that online social support is effective at increasing physical activity in young adults [75]. This review also found that automatic tracking by wearable devices is another common strategy to promote physical activity in older people. Falling and being at risk of falling can in fact be feasibly and accurately detected by wearable devices (e.g., accelerometers and gyroscopes) [76]. Early studies had already shown that fall detectors reduce a person’s fear of falling [77]. Therefore, these strategies should also be embraced in future e-health interventions specifically designed to promote physical activity in older people.

There are several limitations in this review. Most of the control groups in the included studies employed the usual care, but some of them employed an active control. The meta-analysis may have underestimated the effect of this practice. A few randomized controlled trials did not employ parallel groups, leading to uneven group sizes between intervention groups and control groups. This review included a small portion of subjects who are under 60 years old because some trials aimed to recruit older people but they did not specifically exclude people younger than 60 years. More eligible articles may possibly be unincluded if they were not identified by our search strategies.

## Conclusion

E-health interventions are effective at increasing the amount of time spent on physical activity, the energy expended in physical activity, and the number of walking steps. It is recommended that e-health interventions be included in guidelines to enhance physical activity in older people. Walking is the most common form of targeted physical activity promoted in e-health interventions. It is recommended that online social support and automatic tracking (e.g., fall detection and physical activity monitoring) be included in future e-health interventions in order to enhance the effect of those interventions. Further studies should be conducted to examine which e-health strategies are more effective.

## Data Availability

The datasets during and/or analysed during the current study available from the corresponding author on reasonable request.

## Abbreviations

MVPA: Moderate-to-vigorous physical activity
DVD: Digital versatile disc
PDA: Personal digital assistant
CI: Confidence interval
WHO: World Health Organization
